# RNA-sequencing of the *Nyssomyia neivai* sialome: a sand fly-vector from a Brazilian endemic area for tegumentary leishmaniasis and pemphigus foliaceus

**DOI:** 10.1038/s41598-020-74343-y

**Published:** 2020-10-19

**Authors:** Sebastian Vernal, Fabiano Oliveira, Wanderson H. C. Oliveira, Thais M. Goulart, James Oristian, Eric Calvo, Mara C. Pinto, Ana Maria Roselino, José M. C. Ribeiro

**Affiliations:** 1 Division of Dermatology, Department of Clinical Medicine, Ribeirão Preto Medical School, University of São Paulo, Ribeirão Preto, São Paulo Brazil; 2 Laboratory of Malaria and Vector Research, National Institute of Allergy and Infectious Diseases, National Institutes of Health (NIH), Rockville, Maryland USA; 3 Department of Biological Sciences, School of Pharmaceutical Sciences, São Paulo State University (UNESP), Araraquara, São Paulo Brazil; 4 Present Address: Department of Infectious Diseases, University of São Paulo, São Paulo, Brazil

**Keywords:** Infectious-disease diagnostics, Parasitic infection, Sequence annotation

## Abstract

Leishmaniasis encompasses a spectrum of diseases caused by a protozoan belonging to the genus *Leishmania*. The parasite is transmitted by the bite of sand flies, which inoculate the promastigote forms into the host’s skin while acquiring a blood meal. *Nyssomyia neivai* is one of the main vectors of tegumentary leishmaniasis (TL) in Brazil. Southeastern Brazil is an endemic region for TL but also overlaps with an endemic focus for pemphigus foliaceus (PF), also known as *Fogo Selvagem*. Salivary proteins of sand flies, specifically maxadilan and LJM11, have been related to pemphigus etiopathogenesis in the New World, being proposed as an environmental trigger for autoimmunity. We present a comprehensive description of the salivary transcriptome of the *N. neivai*, using deep sequencing achieved by the Illumina protocol. In addition, we highlight the abundances of several *N. neivai* salivary proteins and use phylogenetic analysis to compare with Old- and New-World sand fly salivary proteins. The collection of protein sequences associated with the salivary glands of *N. neivai* can be useful for monitoring vector control strategies as biomarkers of *N. neivai*, as well as driving vector-vaccine design for leishmaniasis. Additionally, this catalog will serve as reference to screen for possible antigenic peptide candidates triggering anti-Desmoglein-1 autoantibodies.

## Introduction

Leishmaniasis encompasses a broad spectrum of diseases caused by an obligate intramacrophage protozoan belonging to the genus *Leishmania* (Kinetoplastida: Trypanosomatidae), occurring predominantly in the tropical and subtropical world regions. The bite of sand fly vectors (Diptera: Psychodidae: Phlebotominae) transmits promastigote forms of *Leishmania* into the host’s skin while acquiring a blood meal^1,2^. Additionally, the phlebotomine deliver the parasite in conjunction with salivary proteins^3^, whose pharmacological activities assist blood feeding by preventing host hemostasis and modulating the host’s immune system^4–6^.

*Leishmania* (*Viannia*) *braziliensis* (Viana 1911) and *Leishmania* (*Leishmania*) *amazonensis* (Lainson & Shaw 1972) are the main species involved in both the cutaneous and mucocutaneous forms of Tegumentary Leishmaniasis (TL) in Brazil^7^. Autochthonous cases of TL have been reported in the northeastern São Paulo state (NSPS), Southeastern Brazil. Both sand flies: *Nyssomyia intermedia* (Lutz & Neiva 1912) (syn = *Lutzomyia (Nyssomyia) intermedia*) and *Nyssomyia neivai* (Pinto 1926) are the main vectors of *L.* (*V*.) *braziliensis* in São Paulo state; however, only *N. neivai* has been recognized in systematic research collections in Northeastern São Paulo State (NSPS) cities^8^.

Pemphigus foliaceus (PF) is an autoimmune bullous disease caused by autoantibodies against desmoglein-1 (Dsg-1). PF is subdivided into classic sporadic worldwide Cazenave’s pemphigus and endemic pemphigus (known in Brazil as *Fogo Selvagem*). Although the pathogenesis of PF remains unclear, genetic and environmental factors have been implicated in the susceptibility to this disease^9,10^. Interestingly, some salivary proteins of sand flies have been associated with pemphigus etiopathogenesis. Maxadilan is a highly immunogenic salivary protein described in *Lutzomyia longipalpis* (Lutz and Neiva 1912), the vector of visceral leishmaniasis (VL) in South America. Higher levels of serum IgG against maxadilan were observed in PF patients compared to healthy controls living in the same endemic region^11,12^. There is also evidence that antibodies raised against LJM11 and LJM17, immunogenic proteins from *Lu. longipalpis*, cross-reacts with antibodies against Dsg-1, and it has been proposed as the antigen that triggers PF diagnosed in Amerindians living in *Mato Grosso* state, Brazil^13,14^. Of note, *Lu. longipalpis* is not widely distributed in NSPS^10,15^.

The profile of salivary components has been defined in ten Old World Phlebotomus species^16–24^, and one Sergentomyia (Sergentomyia schwetzi) species^25^; notwithstanding, the sialotranscriptome of the New World sand flies have been documented only in four species: *Bichromomyia olmeca* (Vargas and Diaz-Najera 1959) (syn = *Nyssomyia olmeca*), *Lutzomyia ayacuchensis* (Caceres and Galati 1988)*, Lu. longipalpis,* and *N. intermedia*^26–29^. Considering the developments on sand fly saliva-based vaccines for *Leishmania sp*. infection, and discovery of possible candidate proteins that might be the trigger of anti-Dsg1 autoantibodies in Brazilian endemic PF in NSPS, we here report the identity and abundances of the putative secreted proteins on the sialome of *N. neivai* by RNA-sequencing. We take this opportunity to compare the most abundant proteins in the *N. neivai* sialome to *N. intermedia*^27^ (the main vector of *Leishmania (V.) braziliensis* in the coastal SP state), and *Lu. longipalpis*^28^ (the vector of VL in *São Paulo* state), besides other published salivary proteins in Old World sand flies.

## Methods

### Collection and maintenance of sand flies

A colony of *N. neivai* was established at *São Paulo* State University^30^ from sand flies collected in Santa Eudóxia, SP state, Brazil (along the edges of the *Mogi Guaçu* River) on the wall of a house, using a manual aspirator between 06:00 PM and 11:00 PM. In the laboratory, the sand flies were maintained in cages covered with voile (30 cm^3^) at 26 ± 1 °C, 80–90% humidity, and a 12:12 (L:D) photoperiod. Salivary glands were dissected as follows: Sand flies were transferred into a tube with mild soap solution. The contents were poured over a fine-mesh screen stretched over a beaker and rinsed with water. We transferred the flies from the mesh screen to a small Petri dish containing 1xPBS. We relocated a freshly rinsed sand fly to a drop of PBS on the microscope slide. The sand flies’ legs and wings were removed. We pierced the thorax and held it against the glass and removed the sand fly’s head. When the head was pulled from the body the salivary glands became visible at the back of the head. The glands were collected with the dissecting pins and transferred to a small, labeled Eppendorf tube for storage. Sand fly identification was based on morphological characteristics of the spermathecae in females and apical genital filaments on males as described by Andrade Filho et al.^31^.

### Salivary gland transportation

Two hundred salivary gland pairs were dissected from starved and non-gravid, 2 to 3 days old *N. neivai* female sand flies. Samples were submitted in 1 mL RNAlater each to the North Carolina State Genomic Sciences Laboratory (Raleigh, NC, USA) for RNA extraction, Illumina library preparation, and sequencing. The salivary glands tissue samples export was approved by the United States Department of Agriculture—Veterinary Permit under the ID number #130339.

### Salivary gland RNA extraction

Prior to extraction, salivary glands were pelleted by the addition of 1 mL PBS in a benchtop centrifuge at 5000×*g* for 10 m to remove the RNAlater. Total RNA was extracted using the RNeasy Mini Kit (Qiagen, MD, USA) following the manufacturer’s protocol for purification of total RNA from animal tissue. Briefly, Qiagen RLT buffer with β-Mercaptoethanol (β-ME) was added to the tissue samples, and samples were homogenized using a Qiagen TissueLyser with 5 mm stainless steel beads (Qiagen). Samples were then purified with provided RNeasy spin columns. Total RNA was then assessed for purity and size integrity using an Agilent 2100 Bioanalyzer with an RNA 6000 Nano Chip (Agilent Technologies, CA, USA). Purification of messenger RNA (mRNA) was performed using oligo-dT beads provided in the NEBNExt Poly(A) mRNA Magnetic Isolation Module (New England Biolabs, MA, USA). Complementary DNA (cDNA) libraries for Illumina sequencing were constructed using the NEBNext Ultra Directional RNA Library Prep Kit (NEB) and NEBNext Multiplex Oligos for Illumina (NEB) using the manufacturer-specified protocol.

### Sialome library

Briefly, the mRNA was chemically fragmented and primed with random Oligos for first strand cDNA synthesis. Second strand cDNA synthesis was then carried out with dUTPs to preserve strand orientation information. The double-stranded cDNA was then purified, end repaired, and “a-tailed” for adaptor ligation. Following ligation, the samples were selected for a final library size (adapters included) of 400–550 bp using sequential AMPure XP bead isolation (Beckman Coulter, USA). Library enrichment was performed, and specific indexes for each sample were added during the protocol-specified PCR amplification. The amplified RNA-seq library fragments were purified and checked for quality and final concentration using an Agilent 2200 Tapestation (D1000 chip, Agilent Technologies, CA, USA) combined with a Qubit fluorometer (Thermo-Fisher, MA, USA). The final quantified libraries were pooled in equimolar amounts for clustering and sequencing on an Illumina HiSeq 2500 DNA sequencer, utilizing a 125 bp single-end cycle sequencing kit (Illumina, CA, USA). The software package Real Time Analysis (RTA) was used to generate raw bcl, or base call files, which were then de-multiplexed by sample into fastq files using bcl2fastq Conversion Software v2.17 (Illumina, CA, USA).

### Bioinformatics

Custom bioinformatic analysis were described elsewhere^32^. Succinctly, low quality reads were trimmed from Fastq files (< 20) and contaminating adapter primer sequences removed. De novo assembly from reads was a result of Abyss^33,34^ (using k parameters from 21 to 91 in fivefold increments) and SOAP de novo-trans^35^ assemblers. The fasta files were combined and further assembled using an iterative blast and CAP3^36^ pipeline as previously described^37^. Coding sequences (CDs) were extracted based on the existence of a signal peptide in the longer open reading frame (ORF) and by similarities to other proteins found in the Refseq invertebrate database from the National Center for Biotechnology Information (NCBI), proteins from “Diptera [organism]” deposited at NCBI’s GenBank, and from Swiss-Prot. Contigs containing an open reading frame and any similarity to sequences in the chosen databases were selected for further analysis. Reads for each library were mapped on the deducted CDs using blastn^38^ with a word size of 25, 1 gap allowed, and 95% identity or better required. Up to five matches were allowed if and only if the scores were the same as the largest score. Mapping of the reads was also included in the Excel spreadsheet. Values of the Reads Per Kilobase of transcript, per Million mapped reads (RPKM)^39^ for each coding sequence were also mapped to a spreadsheet. Automated annotation of proteins was based on a vocabulary of nearly 350 words found in matches to various databases: Swiss-Prot, Gene Ontology, KOG, Pfam, SMART, Refseq-invertebrates, and the GenBank Diptera subset. Raw reads were deposited on the Sequence Read Archive (SRA) of the National Center for Biotechnology Information (NCBI) under BioProject ID PRJNA359206 and read accession SRR5134059. This Transcriptome Shotgun Assembly project has been deposited at DDBJ/EMBL/GenBank under the accession GFDF00000000.

### Phylogenetic analysis

For multiple sequence alignment and phylogenetic analysis, abundant salivary proteins from *N. neivai,* had their predicted signal peptide signal (SignalP-5.0 server^40^) removed, and resulting protein sequence entered into a Basic Local Alignment Search tool (BLAST^38^) against NR and TSA databases. We selected the five most similar homolog sequences (based on the e-value) for each sand fly species. The cut-off to exclude a homologue was an e-value above 1^–10^, except for the *N. neivai* yellow family of proteins where a homologue from *Drosophila* was used to root the tree. Multiple sequence alignment and identity/similarity matrix were constructed on MacVector v15.5.3 with MUSCLE^41^ using PAM 200 profile. We determined the best method for amino acid substitution using the “Find best protein Models” feature of MEGA7^42^. A score was given to each of 56 amino acid substitution models including the mixing Gamma and invariant sites likelihood. The option with the lowest Bayesian information criterion score was selected to build the tree. Through this feature, it was determined that the best amino acid substitution model for phylogeny as follows: WAG for the ML domain and Maxadilan trees. The model WAG with discrete Gamma distribution was used to model evolutionary rate differences among sites (5 categories (+ G, parameter = 1.6114)) for the Yellow proteins tree. For the SP15 family of proteins, the model WAG with a discrete Gamma distribution was used to model evolutionary rate differences among sites (5 categories (+ G, parameter = 4.0108)). The rate variation model allowed for some sites to be evolutionarily invariable ([+ I], 2.61% sites). For the C-type lectin, the best model was LG + F. A discrete Gamma distribution was used to model evolutionary rate differences among sites (5 categories (+ G, parameter = 3.6037)). For Gaps/Missing data treatment, a partial deletion option was utilized. Finally, the reliability of the trees was tested, by bootstrap method (N = 1000).

## Results and discussion

### cDNA library of Nyssomyia neivai salivary gland

cDNA library was constructed from salivary glands of *N. neivai* females dissected up to 3 days after emergence. From this cDNA library 1,302,396 high quality reads were assembled in 1200 contigs (Table 1). Contigs were classified in five categories namely: secreted, housekeeping, transposable elements, viral, and unknown. Remarkably, the secreted proteins category comprised 92.4% of the number of reads, dispersed in 41.2% of the contigs. Most salivary transcriptomes from *Phlebotomus* and *Lutzomyia* genera were based in low output cDNA library sequencing; nevertheless, a high abundance of transcripts encoding secreted proteins were also reported^17–20,23,27,43,44^. Our data further validates the specialization of the salivary gland machinery and the specificity of the material obtained with the sand fly dissection. The housekeeping category had 33.3% of the clusters and only 3.2% of the total sequences. The category of ‘‘unknowns” comprised 24.6% of the clusters and 4.3% of the sequences. Finally, Viral products and transposable elements included less than 1% of the families (0.3% and 0.8% respectively) and less than 0.1% of total sequences. Recently, RNA-seq of salivary glands of Old World *P. kandelakii*^24^ and *Sergentomyia schwetzi* have been published^25^ and the presence of representative salivary proteins has been confirmed.

**Table 1:** Classification of transcripts originating from the sialotranscriptome of *Nyssomyia neivai.*

Class	Number of contigs	% of contigs	RPKM	% RPKM
Secreted	494	41.2	1,203,951	92.4
Housekeeping	399	33.3	42,119	3.2
Unknown	295	24.6	56,243	4.3
Viral products	3	0.3	31	0.0
Transposable elements	9	0.8	52	0.0
Total	1,200	100	1,302,396	100

### Housekeeping and unknown proteins sequences

The 399 clusters (comprising 42,119 sequences) attributed to Housekeeping genes expressed in the salivary glands of *N. neivai* were further divided into 22 subgroups according to their function. Two sets were associated with (a) protein synthesis machinery (22 contigs), including translation, ribosomal structure and biogenesis, and (b) metabolism (94 contigs), a pattern also observed in other sialotranscriptomes. Proteins with unknown function (295 contigs comprising 4.3% of reads) were classified as “unknown”.

### Secreted proteins sequences

The putative secreted salivary proteins of *N. neivai* were classified into 35 main protein families (Table 2). The most abundant transcripts were within the SP13-15 protein family (35.09%), followed by C-type lectins (15.9%), Maxadilan-like (15.6%), ML domain salivary proteins (5.8%), and the Yellow protein family (5.1%). Previously, the novel families 8-kDa, 6-kDa and 5-kDa that were only described in the *N. intermedia* sialome^27^, are now also present in *N. neivai* sialotranscriptome and grouped as Toxin-like peptides.

**Table 2:** Classification of secreted proteins originating from the sialotranscriptome of *Nyssomyia neivai.*

Putative secreted proteins		Number of contigs	% of contigs	RPKM	% RPKM
**Enzymes**	Apyrase	3	0.6	18,214	1.5
	5′ Nucleotidase	4	0.8	7537	0.6
	Hyaluronidase	7	1.4	6887	0.6
	Endonuclease	4	0.8	4927	0.4
	Adenosine deaminase	2	0.4	2923	0.2
	Proteases	53	10.7	1186	0.1
	Lipases*	8	1.6	53	0.0
	Alpha amylase*	1	0.2	2	0.0
Other ubiquitous protein families	Antigen 5 protein	6	1.2	47,405	3.9
	Antimicrobial peptides	8	1.6	781	0.1
	Protease inhibitors	7	1.4	48	0.0
Small molecule binding proteins	C type lectins	20	4.0	190,860	15.9
	ML domain salivary protein	13	2.6	69,404	5.8
	Yellow protein family	13	2.6	61,755	5.1
	Mucins	22	4.5	13,174	1.1
	Diptera conserved salivary secreted peptide	59	11.9	3523	0.3
	JH/PB/OBP	36	7.3	1393	0.1
	Lipocalins	3	0.6	147	0.0
	Hormones	3	0.6	29	0.0
	Galectin	1	0.2	3	0.0
Hematophagous nematocera specific families	D7 family	4	0.8	26,832	2.2
Sand fly specific families	SP13-15 protein family	22	4.4	423,542	35.09
	15 kDa family	15	3.0	278,824	23.2
	14 kDa family	2	0.4	8989	0.7
	13 kDa family	3	0.6	135,729	11.3
	Maxadilan	16	3.2	187,866	15.6
	10 kDa family	3	0.6	40,594	3.4
	Hypothetical secreted protein	149	30.2	31,893	2.6
	Toxin-like peptide	12	2.4	24,330	2.0
	30 kDa family	2	0.4	16,470	1.4
	32.4 salivary protein: Lufaxin	2	0.4	7913	0.7
	34 kDa family	1	0.2	6850	0.6
	Nyssomyia specific	1	0.2	3602	0.3
	38.8 kDa family	1	0.2	1958	0.2
	56.6 kDa salivary protein	3	0.6	1029	0.1
	Gly rich salivary protein	6	1.2	808	0.1
	15.5 kDa family	1	0.2	13	0.0

The following paragraphs describe the most abundant families in detail, focusing on protein family characteristics, possible function, biochemical, immune-modulatory, and antigenic properties: also, phylogenetic analysis in context with related proteins from other Brazilian sand flies and desmoglein proteins.

#### SP15 family

The SP15 is the most abundant secreted protein family in *N. neivai* sialome with 23.13% of the total RPKM in the transcriptome (Table 2). This salivary family is present among all species of sand flies studied so far. In *N. neivai,* we have categorized eight full-length members of this family (Table 3). For further analysis we considered the four most abundant members JAV08233.1, JAV08232.1, JAV08238.1 and JAV08231.1 with 91.37% of the SP13-15 family abundance as highlighted in Table 3.

**Table 3:** SP15 secreted proteins originating from the sialotranscriptome of *Nyssomyia neivai.*

Protein ID	Abundance index (%)	Accession number	Best match to NCBI NR or TSA-NR databases	E-value	Identity (%)	Best match accession number	Seq size	MW	pI
JAV08233.1	26.14	GFDF01005851.1	SP15 family member *N. intermedia*	1.00E−87	90.98	AFP99232.1	133	15.61	9.2
JAV08232.1	23.80	GFDF01005852.1	SP15 family member *N. intermedia*	2.00E−87	90.23	AFP99232.1	133	15.58	9.1
JAV08238.1	23.28	GFDF01005846.1	SP15 family member *N. intermedia*	9.00E−101	98.56	AFP99232.1	139	16.48	9.16
JAV08231.1	18.15	GFDF01005853.1	SP15 family member *N. intermedia*	9.00E−84	90.00	AFP99232.1	158	18.63	9.44
JAV08239.1	5.00	GFDF01005845.1	SP15 family member *N. intermedia*	6.00E−98	97.12	AFP99239.1	139	16.09	9.16
JAV08235.1	1.29	GFDF01005849.1	SP15 family member *N. intermedia*	6.00E−97	97.12	AFP99266.1	139	16.33	9.57
JAV08236.1	1.25	GFDF01005848.1	SP15 family member *N. intermedia*	4.00E−97	96.40	AFP99266.1	139	16.33	9.45
JAV08237.1	1.08	GFDF01005847.1	SP15 family member *N. intermedia*	2.00E−96	97.12	AFP99266.1	139	16.38	9.5

The SP15 family was described for the first time in the sialome *of Lu. longipalpis* as the SL1 family^45^. This family was then also reported in the Old World sand fly *Phlebotomus perniciosus* (Newstead 1911), and later named as SP15 family due to 15-kDa salivary protein from *Phlebotomus papatasi* (Scopoli 1786) (PpSP15: AF335487)^19^. Thus far, SP15-like proteins have only been reported in sand flies and not in any other Dipteran; It has been suggested that SP15-like proteins were derived from an ancestral odorant-binding protein and were closely related to mosquitoes short D7 proteins^16,19^. Alvarenga et al*.*^46^, demonstrated that SP15 from *Phlebotomus duboscqi* (Neveu-Lemaire 1906) inhibit anionic surface-mediated reactions suggesting a role in anticoagulation, inhibiting the activation of FXII and FXI, and anti-inflammatory processes.

PpSP15-like proteins were reported as promising anti-*Leishmania* vaccine candidates. Immunization of mice with *P. papatasi* SP15 protein conferred partial protection against *Leishmania (Leishmania) major* (Yakimoff and Schokhor 1914) infection^43^; furthermore, a DNA vaccine containing the PpSP15 cDNA provided the same protection^43^. ParSP03 (AAX56359), a PpSP15-like protein from *Phlebotomus ariasi* (Tonnoir 1921), elicited similar delayed type hypersensitivity and humoral immune responses upon DNA vaccination^20^. Recently, BALB/c mice immunized to PsSP19 (HM56964), a protein member of the SP15 family from *Phlebotomus sergenti* (Parrot 1917), acted as an adjuvant to accelerate the cell-mediated immune response to co-administered *Leishmania* antigens, providing protection against *Leishmania (Leishmania) tropica* (Wright 1903) infection^47^.

**Figure 1:**
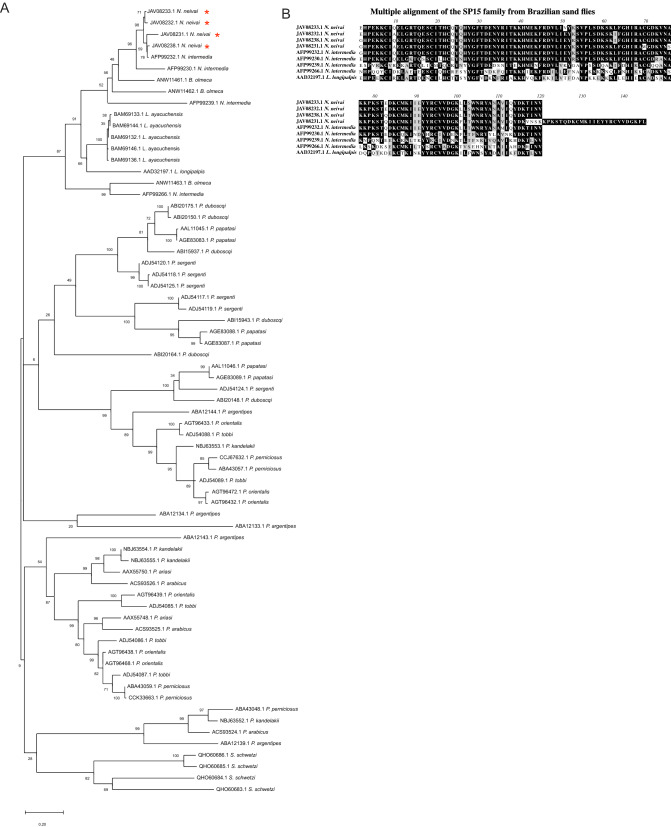
Molecular phylogenetic analysis and sequence alignment of *Nyssomyia neivai* 15 protein family. (**A**) The evolutionary history was inferred by using the Maximum Likelihood method. The tree with the highest log likelihood is shown. The tree is drawn to scale, with branch lengths measured in number of substitutions per site. A discrete Gamma distribution was used to model evolutionary rate differences among sites [5 categories (+ G, parameter = 4.0108)]. The rate variation model allowed for some sites to be evolutionarily invariable ([+ I], 2.61% sites). All positions with less than 95% site coverage were eliminated. Evolutionary analyses were conducted in MEGA7. (**B**) Multiple alignments of SP15 from *Nyssomyia neivai* with *Nyssomyia intermedia* and *Lutzomyia longipalpis* SP15 proteins using Muscle. Black shading represents identical amino acids, light gray shading represents similar amino acids.

Phylogenetic analysis comparing selected sequences from sand fly salivary transcriptomes to the *N. neivai* SP15 family clustered these 4 abundant members closely (Fig. 1A, red asterisks) in a New World sand fly clade next to members of *N. intermedia, B. olmeca*, *Lu. ayacuchensis* and to the only SP-15 family protein described in *Lu. longipalpis* so far, SL1^28^ (AAD32197.1) (Fig. 1A). The remaining clades represent Old world VL and TL vectors and the three members of the *S. schwetzi* SP-15 family. We then aligned *N. neivai* SP15 proteins to SP15 salivary proteins from other sand fly vectors present in Brazil, namely *N. intermedia* and *Lu. longipalpis* (Fig. 1B). *N. neivai* SP15 family proteins shared a relatively high percentage of identity (41.7 to 98.3%) to *N. intermedia* and at a lesser extent to *Lu. longipalpis* SL1 (43.1 to 57.1%) (Table 4).

**Table 4:** Pairwise comparison matrix of identity and similarity percentages.

	JAV08233.*1N. neivai*	JAV08232.*1N. neivai*	JAV08238.*1N. neivai*	JAV08231.*1N. neivai*	AAD32197.*1 L. longipalpis*	AFP99239.*1N. intermedia*	AFP99232.*1N. intermedia*	AFP99230.*1N. intermedia*	AFP99266.*1N. intermedia*
JAV08233.*1 N. neivai*		97.5	95	74.3	57.1	55.5	95.8	81.5	52.9
JAV08232.*1 N. neivai*	98.3		95.8	75	56.3	56.3	95	80.7	52.9
JAV08238.*1 N. neivai*	98.3	98.3		75.7	56.3	54.6	98.3	79.8	52.9
JAV08231.*1 N. neivai*	76.4	77.8	77.8		43.1	41.7	76.4	63.2	41.7
AAD32197.*1 L. longipalpis*	74.8	73.9	73.1	55.6		48.7	56.3	53.8	50.4
AFP99239.*1 N. intermedia*	76.5	75.6	75.6	58.3	67.2		53.8	57.1	40.3
AFP99232.*1 N. intermedia*	98.3	98.3	100	77.8	73.1	75.6		80.7	52.9
AFP99230.*1 N. intermedia*	89.1	89.1	89.1	70.1	71.4	74.8	89.1		47.1
AFP99266.*1 N. intermedia*	72.3	73.1	73.9	58.3	64.7	63.9	73.9	66.4	

*N. neivai* SP15 members shared 76.4 to 98.3% identity (Table 4) with *N. intermedia* Linb-8 (AFP99232.1). Interestingly, BALB/c immunization with DNA plasmids encoding Linb-8 induced the highest humoral immune response against *N. intermedia* salivary gland homogenate (SGH), even greater than Linb-7 (AFP99230.1), another SP-15 protein family member also tested^27^. Linb-7-immunized mice induced a strong humoral response leading to a sustained local inflammatory process, which could exacerbate *Leishmania sp*. infection by *L. (V). braziliensis*^27^. Regarding *N. neivai* and *N. intermedia*, which are the two vectors of *L. (V). braziliensis* in Southeastern Brazil, the observed high similarities between *N. neivai* members and Linb-8 make them possible targets as biomarkers of vector exposure and as a vector-based vaccine for TL in Brazil.

#### SP13 family

**Figure 2:**
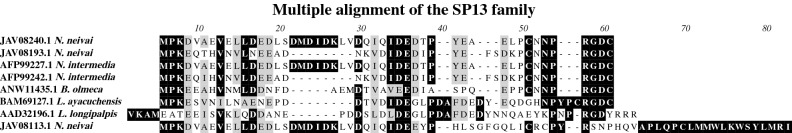
Molecular phylogenetic analysis and sequence alignment of *Nyssomyia neivai* 15 protein family. (**A**) Multiple alignments of SP13 from *Nyssomyia neivai* with other SP13 sand fly salivary proteins using Muscle. Black shading represents identical amino acids, light gray shading represents similar amino acids.

The SP13 *N. neivai* family (JAV08240.1, JAV08113.1 and JAV08193.1) represents 11.26% of the total RPKM in the transcriptome and has the most abundant salivary transcript (JAV08240.1) representing 72.86% of the reads belonging to this family (Table 5). Despite its abundance in *N. neivai*, searches for SP13 subfamily (JAV08240.1, JAV08113.1 and JAV08193.1) related members available in the NCBI NR and TSA-NR databases (blast-P hits with e-value lower that 1e−10), yielded few sand fly salivary proteins. We identified only two members from *N. intermedia* (AFP99227.1 and AFP99242.1), one member for *L. ayacuchensis* (BAM69127.1) and one from *B. olmeca* (ANW11435.1) (Fig. 2)*.*The *N. neivai* JAV08240.1 member it is identical to *N. intermedia* Linb-1 (AFP99227.1) that was also the most abundant contig in the *N. intermedia salivary* transcriptome^27^*.* Interestingly, all the members identified, but JAV08113.1 (Fig. 2) share the RGD domain in the carboxy region that are common in members of the disintegrin family^48,49^. These RGD containing proteins had been previously observed in other New World sand flies such as *Lu. ayacuchensis* (LuayaRGD; BAM69127.1) and *Lu. longipalpis* (LuloRGD; AAD32196). The function and relevance of this family during blood feeding remains to be tested.

**Table 5:** SP13 secreted proteins originating from the sialotranscriptome of *Nyssomyia neivai.*

Protein ID	Abundance index (%)	Accession number	Best match to NCBI NR or TSA-NR databases	E-value	Identity (%)	Best match accession number	Seq size	MW	pI
JAV08240.1	72.86	GFDF01005844.1	SP13 family member *N. intermedia*	6.00E−42	98.53	AFP99227.1	71	8.03	3.86
JAV08113.1	23.73	GFDF01005971.1	SP13 family member *N. intermedia*	2.00E−28	82.09	AFP99227.1	94	10.98	4.49
JAV08193.1	3.41	GFDF01005891.1	SP13 family member *N. intermedia*	1.00E−36	96.72	AFP99242.1	69	7.96	4.57

#### C-type lectin family

**Figure 3:**
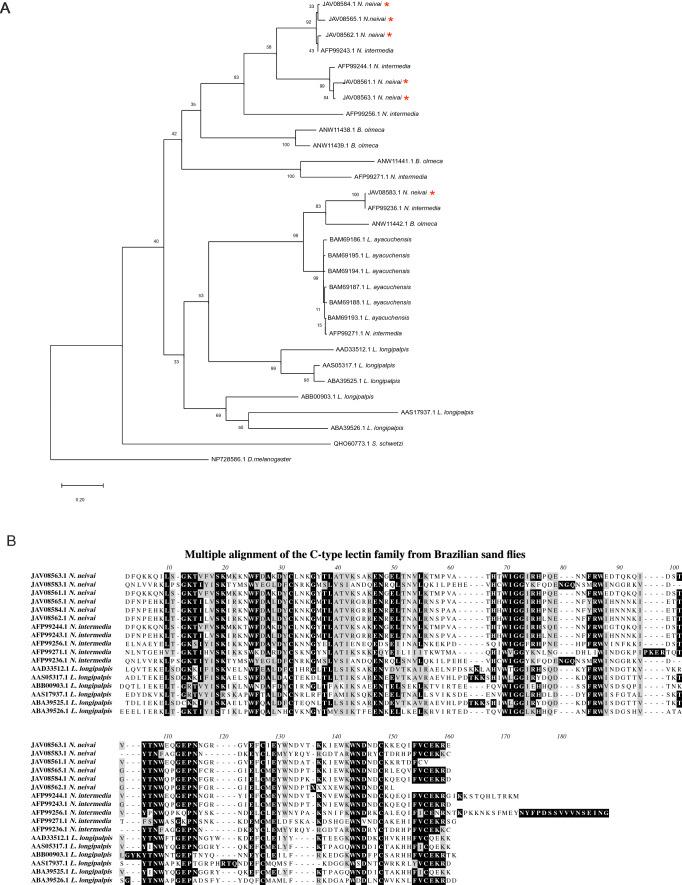
Molecular phylogenetic analysis and sequence alignment of *Nyssomyia neivai* C-type lectin protein family. (**A**) The evolutionary history was inferred by using the Maximum Likelihood method. The tree with the highest log likelihood is shown. A discrete Gamma distribution was used to model evolutionary rate differences among sites [5 categories (+ G, parameter = 3.6037)]. The tree is drawn to scale, with branch lengths measured in number of substitutions per site. All positions with less than 95% site coverage were eliminated. Evolutionary analyses were conducted in MEGA7. (**B**) Multiple alignments of C-type lectins from Brazilian sand flies using Muscle. Black shading represents identical amino acids, light gray shading represents similar amino acids.

We have categorized twelve novel full-length transcripts as C-type lectins in the *N. neivai* sialome, as shown in Table 6. The C-type lectins are the third most abundant salivary family in *N. neivai*. Of those twelve, six members (JAV08563.1, JAV08583.1, JAV08561.1, JAV08565.1, JAV08584.1, JAV08562.1) corresponded to 89.5% of this family abundance and were further considered for in depth analysis (Table 6). In vertebrates, protein-carbohydrate interactions serve multiple functions in the immune system. C-type lectin family members are components of the innate immune response and work via pathogen neutralization through the activation of the complement pathway and adaptive immune response^50^. The C-type lectin putative domain may function as a Ca^2+^-dependent carbohydrate-binding pocket involved in extracellular matrix organization, pathogen recognition, and cell-to-cell interactions^50^. Homologous salivary proteins with molecular weight of 16.2–16.5 kDa have been identified in New World sand flies. Recently, homologues were also found in Old World sand flies by next generation sequencing of salivary glands from *P. kandelakii*^24^, as a partial protein, and *Sergentomyia schwetzi*^25^. The most abundant members of the *N. neivai* C-type lectins family seem to have a close relationship to *N. intermedia* homologues (Fig. 3A)*.* Interestingly, JAV08583.1N*. neivai* protein segregated from the other C-type lectin *N. neivai* members in a subtree that also encompass members from *Lu. ayacuchensis* and *Lu. longipalpis*. The protein sequence alignment comparing Brazilian sand flies depicts a scenario of fast evolution of this family (Fig. 3B), indicated by the large ranges of amino acid identity scores (from 96.4 to 26.7%) across species in pairwise comparisons (Table 7). This may be associated with multiple events of gene duplication and high immune pressure from hosts. The exact role of these proteins in sand flies remains elusive.

**Table 6:** C-type lectin secreted proteins originating from the sialotranscriptome of *Nyssomyia neivai.*

Protein ID	Abundance index (%)	Accession number	Best match to NCBI NR or TSA-NR databases	E-value	Identity (%)	Best match accession number	Seq size	MW	pI
JAV08563.1	19.52	GFDF01005521.1	C-type lectin *N. intermedia*	1.00E−105	92.26	AFP99244.1	156	18.33	8.47
JAV08583.1	15.90	GFDF01005501.1	C-type lectin *N. intermedia*	8.00E−118	100.00	AFP99236.1	157	18.6	8.64
JAV08561.1	15.69	GFDF01005523.1	C-type lectin *N. intermedia*	1.00E−99	90.67	AFP99244.1	152	17.77	8.69
JAV08565.1	14.82	GFDF01005519.1	C-type lectin *N. intermedia*	2.00E−105	94.08	AFP99243.1	158	18.92	8.81
JAV08584.1	13.44	GFDF01005500.1	C-type lectin *N. intermedia*	9.00E−111	96.15	AFP99243.1	156	18.58	9.21
JAV08562.1	10.08	GFDF01005522.1	C-type lectin *N. intermedia*	1.00E−96	95.07	AFP99243.1	149	17.21	8.82
JAV08549.1	3.31	GFDF01005535.1	tfiid subunit *C. appendiculata*	2.00E−85	64.92	JAB55018.1	248	27.68	9.32
JAV08548.1	2.72	GFDF01005536.1	tfiid subunit *C. appendiculata*	2.00E−84	65.29	JAB55018.1	229	25.62	9.1
JAV08581.1	2.41	GFDF01005503.1	C-type lectin *N. intermedia*	3.00E−132	98.37	AFP99256.1	184	21.93	6.18
JAV08559.1	2.10	GFDF01005525.1	C-type lectin *N. intermedia*	2.00E−96	98.54	AFP99271.1	141	16.31	9.37
JAV08596.1	0.01	GFDF01005488.1	C-type lectin *C. tarsalis*	3.00E−156	93.61	JAV29959.1	219	25	8.45
JAV08591.1	0.01	GFDF01005493.1	C-type lectin *P. kandelakii*	6.00E−53	51.37	NBJ58870.1	151	17.41	8.85

**Table 7:** Pairwise comparison matrix of identity and similarity percentages.

	JAV08563.1 N. neivai	JAV08583.1 N. neivai	JAV08561.1 N. neivai	JAV08565.1 N. neivai	JAV08584.1 N. neivai	JAV08562.1 N. neivai	AFP99244.1 N*. intermedia*	AFP99243.1 N*. intermedia*	AFP99256.1 N*. intermedia*	AFP99271.1 N*. intermedia*	AFP99236.1 N*. intermedia*	AAD33512.1 *L. longipalpis*	AAS05317.1 *L. longipalpis*	ABB00903.1 *L. longipalpis*	AAS17937.1 *L. longipalpis*	ABA39525.1 *L. longipalpis*	ABA39526.1 *L. longipalpis*
JAV08563.1N. neivai		33.6	92	65.7	66.4	58	84.6	67.2	40	39.4	33.6	41	39.6	42.4	37.8	36.8	35
JAV08583.1N. neivai	51.7		31.5	36.4	36.4	32.6	31	36.4	26.9	28.4		36.7	32	35.1	30.6	31.3	34.7
JAV08561.1N. neivai	92	48.3		60.6	60.6	59.7	79.2	61.3	36.4	34.5	31.5	39.6	38.9	39.6	34.3	36.1	31.5
JAV08565.1N. neivai	82.5	53.1	75.9		95.6	87.7	58.4	94.9	44.2	38.7	36.4	37.5	37.5	37.5	37.8	34.7	36.4
JAV08584.1N. neivai	82.5	53.1	75.9	97.1		85.5	59.7	96.4	44.2	39.4	36.4	38.2	38.2	37.5	37.8	35.4	36.4
JAV08562.1N. neivai	72.5	47.2	74.6	88.4	87		53.3	88.4	39.8	33.6	32.6	35.9	36.6	33.1	33.3	33.8	31.2
AFP99244.1N*. intermedia*	87.9	49.7	82.6	73.2	73.2	66		62.4	39.4	36.6	31	37.8	37.2	38.1	34.8	35.3	33.1
AFP99243.1N*. intermedia*	82.5	53.8	75.9	97.1	97.1	89.1	75.2		44.2	40.1	36.4	38.9	38.9	37.5	37.8	36.1	36.4
AFP99256.1N*. intermedia*	54.5	42.7	50.3	59.4	57.6	52.4	55.8	57.6		34.3	26.9	29.1	28.5	31.6	27.5	26.7	28.2
AFP99271.1N*. intermedia*	57.7	47.3	52.8	55.6	55.6	49.7	55.6	57.7	46.2		28.4	30.9	32.9	32.4	28.4	29.5	29.9
AFP99236.1N*. intermedia*	51.7	100	48.3	53.1	53.1	47.2	49.7	53.8	42.7	47.3		36.7	32	35.1	30.6	31.3	34.7
AAD33512.1 *L. longipalpis*	58.3	53.7	55.6	59	59	54.5	55.8	59.7	45.3	49	53.7		63.8	42.5	36.8	63.8	35.4
AAS05317.1 *L. longipalpis*	53.5	55.1	50.7	52.1	52.8	47.6	50.6	53.5	42.4	48.3	55.1	77.3		38.4	36.8	92.9	34.7
ABB00903.1 *L. longipalpis*	58.3	54.7	54.9	56.2	56.2	49.7	52.9	55.6	43.9	52	54.7	63	60.3		42.8	37.7	49.3
AAS17937.1 *L. longipalpis*	55.2	49	50.3	55.2	55.9	47.9	49	55.2	40.4	50.7	49	54.2	58.3	57.2		35.4	47.6
ABA39525.1 *L. longipalpis*	54.2	56.5	51.4	52.8	53.5	48.3	50.6	54.2	43	49	56.5	78	96.5	62.3	57.6		36.1
ABA39526.1 *L. longipalpis*	53.8	53.7	49.7	55.2	53.8	46.5	49.4	53.1	42.9	50.3	53.7	59.7	55.6	64.8	62.9	57.6	

#### Maxadilan-like family

Maxadilan (AAA29288.1) is a 7-kDa peptide present in the salivary gland of the sand fly *Lu. longipalpis*. Maxadilan was the first molecule to be identified in sand fly saliva^51^, and it is recognized for its powerful vasodilator effect. *N. neivai* maxadilan-like family corresponds of 11 full length abundantly expressed proteins (Table 8) representing 15.6% of the transcriptome. We will be further discussing 6 members of this family (JAV08475.1, JAV08473.1, JAV08474.1, JAV08472.1, JAV08471.1, JAV08462.1) accounting for 90.14% of this family abundance.

**Table 8:** Maxadilan secreted proteins originating from the sialotranscriptome of *Nyssomyia neivai.*

Protein ID	Abundance index (%)	Accession number	Best match to NCBI NR or TSA-NR databases	E-value	Identity (%)	Best match accession number	Seq size	MW	pI
JAV08475.1	22.83	GFDF01005609.1	Maxadilan related protein *N. intermedia*	1.00E−65	100.00	AFP99229.1	98	10.63	7.76
JAV08473.1	21.44	GFDF01005611.1	Maxadilan related protein *N. intermedia*	3.00E−65	98.98	AFP99229.1	98	10.66	7.76
JAV08474.1	13.14	GFDF01005610.1	Maxadilan related protein *N. intermedia*	4.00E−64	97.96	AFP99229.1	98	10.65	7.76
JAV08472.1	12.54	GFDF01005612.1	Maxadilan related protein *N. intermedia*	4.00E−40	87.32	AFP99237.1	71	8.043	7.71
JAV08471.1	7.84	GFDF01005613.1	Maxadilan related protein *N. intermedia*	3.00E−41	90.14	AFP99237.1	71	8.072	8.53
JAV08462.1	6.44	GFDF01005622.1	Maxadilan *L. longipalpis*	3.00E−07	38.75	P30659.1	79	8.901	9.45
JAV08466.1	5.91	GFDF01005618.1	Maxadilan related protein *N. intermedia*	3.00E−36	83.10	AFP99237.1	71	7.914	8.52
JAV08467.1	4.25	GFDF01005617.1	Maxadilan related protein *N. intermedia*	7.00E−40	88.73	AFP99237.1	71	7.944	7.72
JAV08469.1	2.63	GFDF01005615.1	Maxadilan related protein *N. intermedia*	8.00E−46	97.22	AFP99260.1	72	8.319	8.85
JAV08476.1	2.53	GFDF01005608.1	Maxadilan related protein *N. intermedia*	1.00E−44	97.26	AFP99260.1	73	8.411	8.85
JAV08468.1	0.44	GFDF01005616.1	Maxadilan related protein *N. intermedia*	1.00E−35	81.69	AFP99237.1	71	7.951	9.12

**Figure 4:**
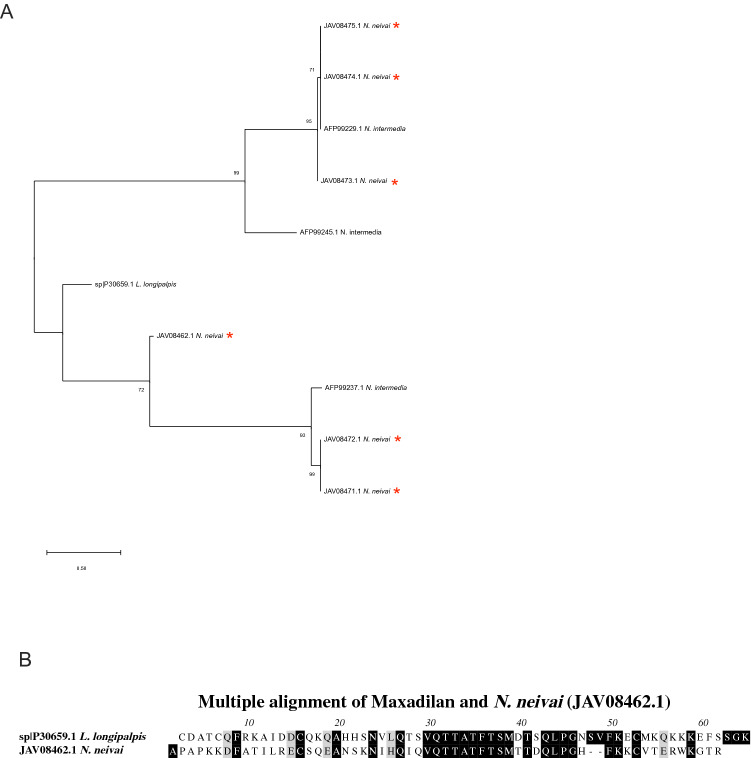
Molecular phylogenetic analysis and sequence alignment of *Nyssomyia neivai* Maxadilan-simile protein family. (**A**) The evolutionary history was inferred by using the Maximum Likelihood method. The tree with the highest log likelihood is shown. The tree is drawn to scale, with branch lengths measured in number of substitutions per site. All positions with less than 95% site coverage were eliminated. Evolutionary analyses were conducted in MEGA7. (**B**) Multiple alignments of Maxadilan and *N. neivai (JAV08462.1)* proteins using Muscle. Black shading represents identical amino acids, light gray shading represents similar amino acids.

Comparative analyses of abundant transcripts from *N. neivai* maxadilan-like family were able to identify three homologues in *N. intermedia* (Fig. 4A)*.* Phylogenetic topology shows that a main clade clustered the most abundant *N. neivai* members (JAV08475.1, JAV08473.1, JAV08474.1) with Linb-9 (AFP99245.1), while the other *N. neivai* members clustered with Linb-25 from *N. intermedia*. Maxadilan has its own branch and, JAV08462.1 from *N. neivai* was the closest relative to Maxadilan with 37.5% identity and 56.2% similarity suggesting that *N. neivai* JAV08462.1 (Fig. 4B) may have preserved its pharmacological properties^52^. *N. neivai* JAV08462.1 represents the sixth most abundant member with 6.44% of the maxadilan-like family abundance. Linb-147 (JK846521), a partial sequence from *N. intermedia,* showed a similar match to maxadilan, provided for only 34% identity and 70% similarity over a stretch of 50 amino acids. This sequence seems to be scarcely present in *N. intermedia* sialome, with only one transcript identified in its cDNA library, as compared to 30 transcripts of maxadilan present in *Lu. longipalpis* sialome^27,28^.

Maxadilan-like proteins have never been identified in Old World *Phlebotomus* species^17–20,23,27,43^. *Phlebotomus* sand flies, except for *P. duboscqi*, contain large amounts of vasodilatory adenosine and AMP in their saliva^18–20,23,25^. Interestingly, they lack adenosine deaminase (ADA), an enzyme that hydrolyzes adenosine and adenosine monophosphate (AMP)^53,54^. In contrast, *Lu. longipalpis* has ADA and lacks adenosine and AMP in the saliva^28,45^. Unexpectedly, neither ADA nor maxadilan were identified in *Lu. ayacuchensis*. Thus, with those exceptions in general, salivary vasodilators of sand flies are adenosine and AMP in the *Phlebotomus* complex, and maxadilan in the *Lu. longipalpis* complex.

Inoculation of maxadilan in experimental animals exacerbates *Leishmania* infection to the same degree as the whole salivary gland homogenate^55^. This peptide can drive a Th1 response to Th2, up-regulate IL-10 and TGF-β production, and suppress IL-12p40, TNF-α, and NO production^56,57^.

In animal models, mice vaccinated with maxadilan become markedly protected against *Leishmania* infection, producing not only anti-maxadilan antibodies, but also immune CD4 + T cells specific to maxadilan generating IFN-γ and inducing NO production^55^. Notoriously, immunization against maxadilan also inhibits blood meal acquisition by sand flies, a promising target to block the vector reproductive process^58^. Nonetheless, maxadilan is not free of polymorphisms, its amino acid substitution rate is around 23%, with most amino acids positions not being conserved among homologues^59,60^. Considering *Lu. longipalpis* as the vector of VL and *N. neivai* as the vector of TL in the same endemic Brazilian regions^8,61^, maxadilan-like proteins could bring a new insight for a common vaccine targeting VL and TL.

#### ML domain peptide family

We have categorized 10 full length abundant proteins as being part of the ML domain family that mapped to 5.8% of the *N. neivai* transcriptome (Table 9). We focused on the six most abundant members (JAV08576.1, JAV08588.1, JAV08582.1, JAV08586.1, JAV08585.1, JAV08572.1) for a comparative analysis representing 88.01% of this family representativity (Table 9). The MD-2-related lipid-recognition (ML) domain is implicated in lipid-mediated membrane binding mechanisms with a role in the execution and regulation of many cellular processes, including cell signaling and membrane trafficking^62^. The ML domain from the SMART database hints that this proteins may be involved in innate immunity or as an antagonist of lipid mediators of hemostasis and inflammation^63^.

**Table 9:** ML domain secreted proteins originating from the sialotranscriptome of *Nyssomyia neivai.*

Protein ID	Abundance index (%)	Accession number	Best match to NCBI NR or TSA-NR databases	E-value	Identity (%)	Best match accession number	Seq size	MW	pI
JAV08576.1	19.01	GFDF01005508.1	Putative ML domain salivary peptide *C. appendiculata*	5.00E−112	87.06	JAB54888.1	170	19.90	7.71
JAV08588.1	18.94	GFDF01005496.1	Putative ML domain salivary peptide *C. appendiculata*	5.00E−112	86.55	JAB54888.1	171	20.00	7.55
JAV08582.1	16.91	GFDF01005502.1	ML domain salivary peptide *N. intermedia*	2.00E−105	98.01	AFP99241.1	151	16.60	9.51
JAV08586.1	13.53	GFDF01005498.1	ML domain salivary peptide *N. intermedia*	3.00E−109	98.71	AFP99248.1	155	17.25	8.47
JAV08585.1	13.49	GFDF01005499.1	ML domain salivary peptide *N. intermedia*	1.00E−109	99.35	AFP99248.1	155	17.20	8.47
JAV08572.1	6.22	GFDF01005512.1	ML domain salivary peptide *N. intermedia*	4.00E−112	98.72	AFP99259.1	165	18.99	9.39
JAV08554.1	3.36	GFDF01005530.1	ML domain salivary peptide *N. intermedia*	4.00E−88	100.00	AFP99264.1	124	14.41	8.99
JAV08568.1	3.25	GFDF01005516.1	LolMLc *B. olmeca*	7.00E−65	57.74	ANW11448.1	165	19.16	9.45
JAV08580.1	2.80	GFDF01005504.1	ML domain salivary peptide *N. intermedia*	5.00E−114	98.73	AFP99264.1	157	18.12	8.87
JAV08567.1	2.49	GFDF01005517.1	LolMLc *B. olmeca*	9.00E−65	57.74	ANW11448.1	165	19.12	9.45

**Figure 5:**
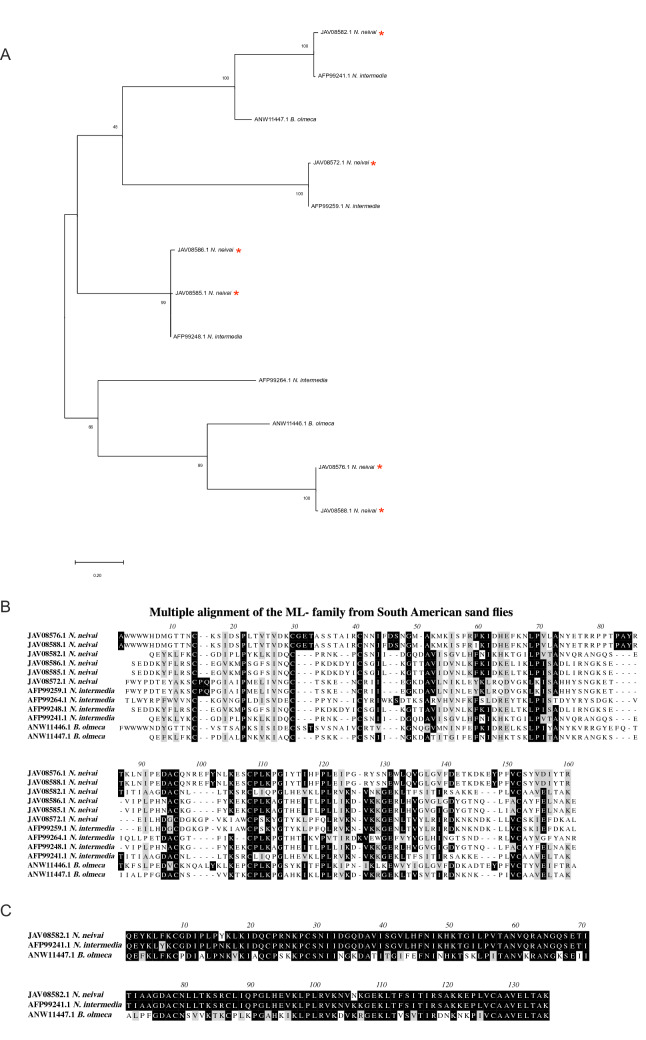
Molecular phylogenetic analysis and sequence alignment of ML-domain protein family of *Nyssomyia neivai.* (**A**) The evolutionary history was inferred by using the Maximum Likelihood method. The tree with the highest log likelihood is shown. The tree is drawn to scale, with branch lengths measured in number of substitutions per site. All positions with less than 95% site coverage were eliminated. Evolutionary analyses were conducted in MEGA7. (**B**) Multiple alignments of ML-domain from *Nyssomyia neivai* with South American sand flies ML-domain proteins using Muscle. Black shading represents identical amino acids, light gray shading represents similar amino acids. (**C)** Alignment of *Nyssomyia neivai* JAV08582.1, *N. intermedia* AFP99241.1 and *B. olmeca* ANW11447.1 from the ML-domain protein family using Muscle. Black shading represents identical amino acids, light gray shading represents similar amino acids.

This family is relatively common in tick sialomes^63^ but it had only been described in *N. intermedia* and *B. olmeca* sialomes so far^27,29^. *N. neivai* phylogenetic analysis indicates the presence of 3 subfamilies with the ML domain, with several likely events of gene duplication occurring in *N. neivai* (Fig. 5A)*.* ML domain salivary proteins present in sand flies seems to be a very divergent family with few conserved amino acids across species (Fig. 5B); however, when comparing the clustered molecules within each of the clades we start to appreciate a higher degree of conservation(Fig. 5C), for example comparing the *N. intermedia* ML family sequence AFP99241.1 and *B. olmeca* (ANW11447) that share the same subtree with *N. neivai* JAV08582.1 in Fig. 5A, we observe 97.8% and 64.9% identity (Fig. 5C), respectively, likely hinting at possibly three independent proteins families within the ML domain.

#### Yellow protein family

*N. Neivai* salivary transcriptome encompasses eight members of the yellow salivary proteins corresponding to 5.1% of the total sialome. From these eight members three of them corresponds to 99.72% of the yellow family abundance (Table 10).

**Table 10:** Yellow-related secreted proteins originating from the sialotranscriptome of *Nyssomyia neivai.*

Protein ID	Abundance index (%)	Accession number	Best match to NCBI NR or TSA-NR databases	E-value	Identity (%)	Best match accession number	Seq size	MW	pI
JAV07960.1	36.18	GFDF01006124.1	Hypothetical protein *L. ayacuchensis*	0	76.94	BAM69111.1	398	45.44	9.49
JAV07959.1	34.61	GFDF01006125.1	Hypothetical protein *L. ayacuchensis*	0	76.69	BAM69111.1	398	45.40	9.47
JAV07968.1	28.94	GFDF01006116.1	Yellow-related salivary protein *N. intermedia*	0	98.03	AFP99235.1	406	46.13	8.64
JAV07963.1	0.13	GFDF01006121.1	43 kDa salivary yellow-related protein SP04 *P. argentipes*	5.00E−25	24.72	ABA12136.1	408	47.19	6.39
JAV07967.1	0.05	GFDF01006117.1	Putative major royal jelly protein *P. kandelakii*	0	76.00	NBJ60408.1	447	49.99	6.89
JAV07966.1	0.05	GFDF01006118.1	Putative major royal jelly protein *P. kandelakii*	0	76.00	NBJ60408.1	460	51.49	6.83
JAV07964.1	0.05	GFDF01006120.1	Major royal jelly protein *P. kandelakii*	4.00E−33	27.17	NBJ63551.1	450	51.58	5.95
JAV07955.1	0.00	GFDF01006129.1	AAEL011699-PA *A. aegypti*	0	62.69	EAT36216.1	490	55.03	5.52

Yellow-related proteins are abundantly expressed in salivary glands of phlebotomies, mainly in Old World sand flies^17–20,23–25,27,43,44^. The Yellow family was the most abundant salivary protein family detected, also by next generation sequencing, on *Phlebotomus kandelakii* salivary glands (accounting to 31.7% of the mRNA on salivary glands)^24^ contrasting with the limited presence of this family in *N. neivai* (5.1%). Phlebotomine yellow-related proteins are characterized by having the major royal jelly protein domain (MRJP). Originally, MRJP proteins were described from honeybee larval jelly, making up to 90% of the protein content^64^. Sequences related to MRJP proteins were described in *Drosophila*, where it is related to cuticle pigmentation and, when mutated, it produced a yellow phenotype and thus named Yellow proteins^65^. It was later found that in Diptera they had a dopachrome oxidase function^66,67^.

In bloodsucking Diptera, salivary yellow-related proteins have only been described in sand flies (all sand fly species studied to date)^17–20,23–25,27,43,44^, and Glossina morsitans morsitans (Westwood 1851)^68^. The proteins of this family are immunogenic and host antibody responses to this protein can be a potential marker for sand fly exposure in experimentally bitten mice and dogs, as well as naturally exposed dogs, humans, and foxes^3,69^. *Lu. longipalpis* proteins, LJM11, LJM111 and LJM17, act as high affinity binders of pro-inflammatory biogenic amines such as serotonin, catecholamines, and histamine, suggesting that the proteins play a role for the reduction of inflammation during sand fly blood-feeding^3,70^, this activity has also been confirmed in salivary yellows from Old World *P. orientalis* and *P. perniciosus*^71,72^.

A combination of recombinant LJM17 and LJM11 successfully substituted *Lu. longipalpis* whole salivary gland homogenate in probing sera of individuals for vector exposure^73^. Yellow proteins are also under consideration for anti-*Leishmania* vector-based vaccines. LJM17 from *Lu. longipalpis* elicited leishmanicidal Th1 cytokines in immunized dogs^74,75^, and LJM11 protected laboratory animals against *L. (L.) infantum* (Nicolle 1908), *L. (L.) major,* and *L. (V.) braziliensis*^70,76,77^.

In contrast, mice immunized with *P. papatasi* yellow-related proteins PpSP42 or PpSP44 (AAL11052 and AAL11051, respectively) elicited Th2 cytokines and exacerbated *L. (L.) major* infection^78^. Other yellow-related proteins from *P. papatasi*, specifically PPTSP44 (AGE83095.1), induced a strong Th1 response constituting a potential vaccine candidates against leishmaniasis^79^. It remains to be elucidated whether the protection induced by yellow-related proteins is related to particular protein immunogenicity, to sand fly species, or to the vector-*Leishmania* host combination, as all of these factors can contribute to vaccine efficacy. New approaches using novel vaccine techniques, consisting of a single dose of plasmid, followed by two doses of recombinant Canarypoxvirus expressing *Lu. longipalpis* yellow-related salivary proteins, are a promising strategy to control *Leishmania* infection^75^.

**Figure 6:**
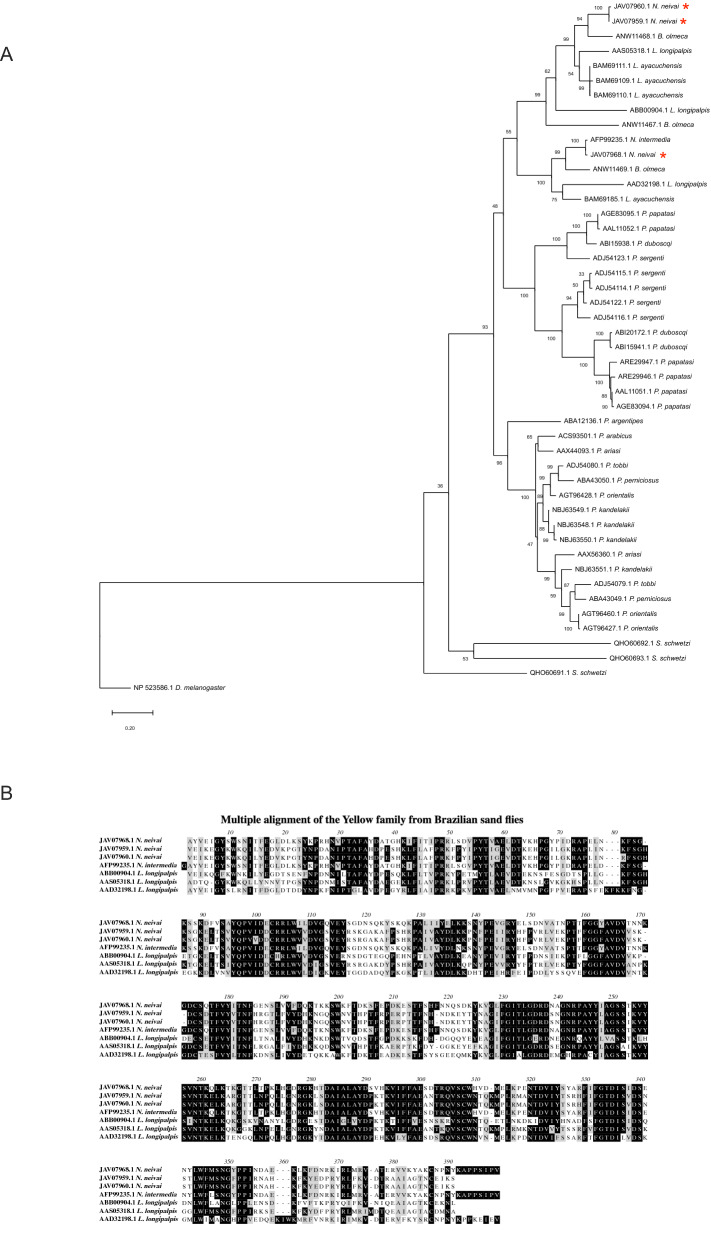
Molecular phylogenetic analysis and sequence alignment Yellow-related protein family of *Nyssomyia neivai*. (**A**) The evolutionary history was inferred by using the Maximum Likelihood method. The tree with the highest log likelihood is shown. The tree is drawn to scale, with branch lengths measured in number of substitutions per site. A discrete Gamma distribution was used to model evolutionary rate differences among sites [5 categories (+ G, parameter = 1.6114)]. All positions with less than 95% site coverage were eliminated. Evolutionary analyses were conducted in MEGA7. (**B**) Multiple alignments of Yellow-related protein from *Nyssomyia neivai* with *Nyssomyia intermedia* and *Lutzomyia* longipalpis Yellow-related protein using Muscle. Black shading represents identical amino acids, light gray shading represents similar amino acids.

Phylogenetic analysis segregated the New World sand fly yellow proteins in its own clade separated from VL and TL Old World sand fly yellow proteins (Fig. 6A). The New World sand fly yellow proteins clade branched out in two subclades, one with the presence of *Lu. longipalpis* LJM11(AAS05318.1) and LJM111(ABB00904.1) yellow proteins that clustered with *N. neivai* yellow-related protein JAV07960.1 and JAV07959.1 (Fig. 6A). Of note, we can observe that these two *N. neivai* yellows are closely related to the yellow from *B. olmeca* (ANW11468.1) (Fig. 6A). In the other subclade *N. neivai* yellow-related protein JAV07968.1 clustered closely with the *N. intermedia* (AFP99235.1) and with the *Lu. longipalpis* LJM17 (AAD32198.1) member (Fig. 6A). Multiple alignment of *N. neivai, Lu. longipalpis,* and *N. intermedia* (Fig. 6B) shows a high level of conservation among these proteins. For example, *N. neivai* JAV07960.1 and *Lu. longipalpis* LJM11(AAS05318.1) share 73.3% identity and 84.6% similarity). In the other hand, *N. neivai* yellow-related protein JAV07968.1 share a closer relationship with *N. intermedia* Linb-21 (AFP99235.1) highlighted by its 97.7% identity compared to 62.4% identity with *Lu. longipalpis* LJM17 (AAD32198.1).

### Sand flies’ salivary proteins and pemphigus foliaceus

Sand fly salivary proteins have been associated with endemic PF pathogenesis in Brazil and Tunisia^10–12,52,80–82^. Screening of sera from pemphigus foliaceus patients from NSPS antibodies against *Lu. longipalpis* maxadilan^11^ while Mato Grosso do Sul State patients reacted to *Lu. longipalpis* LJM11^13^ and LJM17^14^. Similarly, Tunisian PF patients also reacted to several salivary proteins from *P. papatasi*^80^.

The mechanisms through which exposure to sand fly bites may induce an autoimmunity triggering production of IgG autoantibodies against Dsg1 in genetically susceptible individuals remains to be teased out. In fact, IgG antibodies against salivary homogenates from *N. neivai* correlated positively with IgG anti-Dsg1 in PF patients^10^. An antigenic cross-reactivity between salivary proteins and Dsg1 is the most plausible hypothesis for anti-Dsg1 autoantibodies production following sand fly bites. Nevertheless, both BLAST and PSIBLAST do not detect any highly significant homology between *N. neivai*, *Lu. longipalpis* or *P. papatasi* salivary proteins and Dsg1^14^. Other pathogenic mechanisms that could explain the loss of Dsg1 self-antigen tolerance induced by exposure to sand fly salivary proteins remains to be tested.

The presence of an abundant maxadilan-simile transcripts in *N. neivai* (JAV08462.1) sialome may explain the antibody reactivity to maxadilan in NSPS patients, where *N*. *neivai* but no *Lu*. *longipalpis* is present^9^. Further testing with a *N. neivai* recombinant maxadilan can help confirm this assumption. Moreover, testing the PF sera from NSPS against recombinant yellow proteins identified in *N. neivai* would also be desirable in our PF casuistic.

Considering that non-homologous salivary proteins from different sand fly species were associated with PF pathogenesis^10–14,81,82^, we may expect that not a single peptide may act as an independent antigen to trigger PF. More than one protein may be involved in the PF pathogenesis considering the shared pharmacological properties and conformational mimotopes of these proteins in distinct biting sand fly species.

## Conclusion

Leishmaniasis is still a frequent and neglected disease in Brazil. Our results add valuable data related to New World Phlebotomine salivary proteins, expanding the findings reported in *Lu. longipalpis* and *N. intermedia* sialomes. The availability of the identity of the most abundant *N. neivai* salivary proteins of the three main species of sand flies widely distributed in Brazil will bring new insights into the host-vector-parasite relationship of *L. (L.) infantum* and *L. (V.) braziliensis* infections and may point to targets of interest for a vector-based vaccine. We hope the availability of this compilation of *N. neivai* salivary proteins by abundance can inform researchers on the selection *N. neivai* candidates for future experiments*.* Production of distinct abundant *N. neivai* recombinant proteins can be used to test individual candidates for the etiology of PF as the trigger of anti-Dsg1 autoantibodies, and also used as biomarkers of vector exposure translating into monitoring tools for vector intervention campaigns.

## Data Availability

All relevant data are within the paper.
